# *ACYP2* polymorphisms are associated with the risk of liver cancer in a Han Chinese population

**DOI:** 10.18632/oncotarget.18574

**Published:** 2017-06-19

**Authors:** Zhong Chen, Yu Sun, Zhenxiong Xu, Junnv Xu, Jingjie Li, Mengdan Yan, Jing Li, Tianbo Jin, Haifeng Lin

**Affiliations:** ^1^ Department of Thoracic Surgery, Second People's Hospital of Hainan Province, Hainan, 572200, China; ^2^ Department of General Surgery, Second People's Hospital of Hainan Province, Hainan, 572200, China; ^3^ Department of Internal Medicine-Oncology, Agricultural Reclamation General Hospital of Hainan Province, Hainan, 570311, China; ^4^ Key Laboratory of Resource Biology and Biotechnology in Western China, Northwest University, Ministry of Education, School of Life Sciences, Northwest University, Shaanxi, 710069, China; ^5^ Xi’an Tiangen Precision Medical Institute, Shaanxi, 710075, China

**Keywords:** ACYP2, polymorphism, liver cancer, case-control, Han Chinese population

## Abstract

We explored the association between single nucleotide polymorphisms (SNPs) in *ACYP2* and liver cancer risk. Thirteen SNPs were genotyped in 473 cases and 564 controls. Genetic model, linkage disequilibrium, and haplotype analyses were performed to evaluate the association between *ACPY2* SNPs and liver cancer risk. We found that rs6713088 (G allele: odds ratio [OR] = 1.27, 95% confidence interval [CI]: 1.07−1.52, *P* = 0.007; GG vs. CC: OR = 1.49, 95% CI: 1.02−2.1, *P* = 0.038), rs843711 (T allele: OR = 1.29, 95% CI: 1.09−1.54, *P* = 0.004; TT vs. CC: OR = 1.62, 95% CI: 1.13−2.31, *P* = 0.008), rs843706 (A allele: OR = 1.30, 95% CI: 1.09−1.55, *P* = 0.003; AA vs. CC: OR = 1.62, 95% CI: 1.13−2.31, *P* = 0.008), and rs843645 (GG vs. AG: OR = 1.40, 95% CI: 1.07−1.82, *P* = 0.014) were associated with an increased risk of liver cancer. In contrast, rs1682111 (A allele: OR = 0.77, 95% CI: 0.640−0.94, *P* = 0.007; AT vs. TT: OR = 0.69, 95% CI: 0.53−0.91, *P* = 0.007), rs843720 (additive model: OR = 0.82, 95% CI: 0.68−1.00, *P* = 0.049), ATATCGCC and CG haplotypes (OR = 0.76, 95% CI: 0.62−0.92, *P* = 0.006; OR = 0.78, 95% CI: 0.65−0.93, *P* = 0.006, respectively) were significantly decreased liver cancer risk. Our results confirmed that rs6713088, rs843645, rs843711 and rs843706 were significantly increased liver cancer risk, but rs1682111, rs843720 and haplotypes (ATATCGCC and CG) were significantly decreased liver cancer risk in a Han Chinese population.

## INTRODUCTION

Liver cancer is the second leading cause of cancer-related death among men worldwide. There were an estimated 782,500 new liver cancer cases and 745,500 liver cancer-related deaths in 2012 worldwide. China alone accounted for approximately 50% of the total number of cases and deaths [1]. Liver cancer is a complex and multifactorial disease regulated by both genetic and environmental factors. Epidemiological studies have shown that the major environmental risk factors for liver cancer include chronic Hepatitis B virus (HBV) and Hepatitis C virus (HCV) infection, dietary aflatoxin exposure, alcohol consumption, cigarette smoking, obesity, diabetes, and iron overload [2, 3]. However, not all individuals with exposure to these risk factors develop liver cancer. Hepatocellular carcinoma (HCC) accounts for between 70% and 85% of primary liver cancers. Recently, some Genome-wide association studies (GWAS) have identified several loci associated with the risk of HCC, such as SNPs in the gene *KIF1B*, *MICA*, *HLA-DQA/DQB*, and *GRIK1*, respectively [4–6].

Acylphosphatase 2 (*ACYP2*) encodes a small cytosolic enzyme (11 kDa) that is widely expressed in vertebrates. Two isoenzymes have been identified (muscle and erythrocyte) that share 56% sequence identity [7]. *ACYP2* hydrolyzes the phosphoenzyme intermediate of various membrane pumps such as the Ca^2+^/Mg^2+^-ATPase from the sarcoplasmic reticulum of skeletal muscle [8]. Overexpression of *ACYP2* induces cell differentiation [9] and apoptosis [10]. In addition, a genome wide association study demonstrated an association between *ACYP2* polymorphisms and telomere length [11].

Interestingly, telomere length was also correlated with risk of liver cancer [12, 13]. However, a direct association between single nucleotide polymorphisms (SNPs) in *ACYP2* and susceptibility to liver cancer has not been established. We hypothesized that genetic variations in *ACYP2* could influence susceptibility to liver cancer. We selected 13 SNPs from the HapMap databases with a minor allele frequency (MAF) > 5% in the Chinese Han population, and designed a case-control study to investigate the associations between these SNPs and the risk of liver cancer.

## RESULTS

### Study population

The characteristics of all study participants are shown in Table 1. There were 473 liver cancer patients (390 men and 83 women) and 564 healthy controls (339 men and 225 women) included in the study. The average ages of the cases and controls were 55.8 and 53.9 years, respectively. There were significant differences in the age and gender distribution between the cases and controls (*P* < 0.05) (Table 1). Therefore, we adjusted for these variables in the multivariate, unconditional logistic regression.

**Table 1:** Characteristics of the cases and controls

Variables	Cases (*n* = 473)	Controls (*n* = 564)	*P*
Sex			**< 0.001**
Male	390 (82.5%)	339 (60.1%)	
Female	83 (17.5%)	225 (39.9%)	
Age			**0.010**
yrs (mean ± SD)	55.8 ± 12.2	53.9 ± 11.5	

SD: Standard deviation.

*P* < 0.05 indicates statistical significance.

The sequences of the primers that were used to genotype each SNP are shown in Supplementary Table 1. The average SNP call rate was 98.3% in cases and controls. The allele distributions and MAFs of all SNPs and the results of Hardy-Weinberg equilibrium (HWE) tests are shown in Table 2. The genotype distributions of the 13 SNPs among the controls were compatible with HWE (all *P* > 0.05). The G allele of rs6713088, T allele of rs843711, and A allele of rs843706 were associated with a 1.27-fold, 1.29-fold, and 1.30-fold increased risk of liver cancer, respectively (odds ratio [OR] = 1.27, 95% confidence interval [CI]: 1.07–1.52, *P* = 0.007; OR = 1.29, 95% CI: 1.09–1.54, *P* = 0.004; OR = 1.30, 95% CI: 1.09–1.55, *P* = 0.003, respectively). In contrast, the A allele of rs1682111 was associated with a decreased risk of liver cancer (OR = 0.77, 95% CI: 0.64–0.94, *P* = 0.007). However, no significant association between SNPs in *ACYP2* and liver cancer risk was observed after Bonferroni correction.

**Table 2:** Allele frequencies distribution and association with the risk of liver cancer

SNP-ID	Chromosome	Position	Band	Role	Allele (A/B)	MAF		HWE *P*^a^	OR	95% CI	*P*^b^
						Cases	Controls				
rs6713088	2	54345469	2p16.2	Intron	G/C	0.452	0.393	0.379	1.27	1.07–1.52	0.007
rs12621038	2	54391113	2p16.2	Intron	T/C	0.445	0.440	0.608	1.02	0.86–1.22	0.813
rs1682111	2	54427979	2p16.2	Intron	A/T	0.275	0.329	0.775	0.77	0.64–0.94	0.008
rs843752	2	54446587	2p16.2	Intron	G/T	0.296	0.266	0.518	1.16	0.95–1.40	0.142
rs10439478	2	54459450	2p16.2	Intron	C/A	0.427	0.402	0.382	1.11	0.93–1.32	0.258
rs17045754	2	54496757	2p16.2	Intron	C/G	0.197	0.167	0.761	1.22	0.98–1.53	0.077
rs843720	2	54510660	2p16.2	Intron	G/T	0.303	0.342	0.779	0.84	0.69–1.01	0.057
rs843645	2	54474664	2p16.2	Downstream	G/T	0.282	0.252	0.263	1.17	0.96–1.42	0.116
rs11125529	2	54475866	2p16.2	Downstream	A/C	0.185	0.164	0.644	1.16	0.92–1.46	0.201
rs12615793	2	54475914	2p16.2	Downstream	A/G	0.201	0.178	0.315	1.16	0.93–1.45	0.181
rs843711	2	54479117	2p16.2	Downstream	T/C	0.501	0.437	1.000	1.29	1.09–1.54	0.004
rs11896604	2	54479199	2p16.2	Downstream	G/C	0.214	0.185	0.675	1.20	0.97–1.49	0.098
rs843706	2	54480369	2p16.2	3′ UTR	A/C	0.504	0.439	1.000	1.30	1.09–1.55	0.003

*P*^a^-values were calculated using Fisher's exact test.

*P*^b^-values were calculated using Pearson's chi-square test.

*P* < 0.05 indicates statistical significance.

Significant associations between *ACPY2* genotypes and the risk of liver cancer are shown in Table 3. We found that rs6713088, rs843645, rs843711, and rs843706 were associated with an increased risk of liver cancer even after adjustment for age and gender (GG vs. CC: OR = 1.49, 95% CI: 1.02–2.1, *P* = 0.038; GT vs. TT: OR = 1.40, 95% CI: 1.07–1.82, *P* = 0.014; TT vs. CC: OR = 1.62, 95% CI: 1.13–2.31, *P* = 0.008; AA vs. CC: OR = 1.62, 95% CI: 1.13–2.31, *P* = 0.008, respectively). In contrast, the AT genotype of rs1682111 was associated with a decreased risk of liver cancer compared to the TT genotype (OR = 0.69, 95% CI: 0.53–0.91, *P* = 0.007, respectively). Interestingly, the AA genotype of rs1682111 was also associated with a decreased risk of liver cancer compared to the TT genotype after adjustment for age and gender (OR = 0.62, 95% CI: 0.39–0.98, *P* = 0.039). We also found that the GG genotype of rs843720 was associated with a decreased risk of liver cancer compared to the TT genotype without adjustment for age and gender (OR = 0.64, 95% CI: 0.41–1.00, *P* = 0.048).

**Table 3:** Genotype frequencies distribution and association with liver risk cancer

SNP-ID	Genotype	Cases	Controls	Without adjustment			With adjustment		
				OR	95% CI	*P*^a^	OR	95% CI	*P*^b^
	CC	138	202	1.00	–		1.00	–	
rs6713088	GC	242	279	1.27	0.96–1.67	0.091	1.27	0.95–1.69	0.100
	GG	93	82	1.66	1.15–2.40	**0.007**	1.49	1.02–2.18	**0.038**
	CC	139	180	1.00	–		1.00	–	
rs12621038	TC	245	271	1.17	0.88–1.55	0.271	1.28	0.96–1.71	0.098
	TT	87	112	1.01	0.70–1.44	0.974	1.08	0.75–1.56	0.685
	TT	251	252	1.00	–		1.00	–	
rs1682111	AT	181	253	0.72	0.55–0.93	**0.012**	0.69	0.53–0.91	**0.007**
	AA	39	59	0.66	0.43–1.03	0.068	0.62	0.39–0.98	**0.039**
	TT	232	306	1.00	–		1.00	–	
rs843752	GT	201	214	1.24	0.96–1.60	0.103	1.23	0.94–1.61	0.127
	GG	39	43	1.20	0.75–1.91	0.451	1.13	0.70–1.83	0.612
	AA	154	206	1.00	–	–	1.00	–	–
rs10439478	CA	233	261	1.19	0.91–1.57	0.204	1.20	0.91–1.60	0.201
	CC	85	96	1.18	0.83–1.70	0.355	1.31	0.90–1.90	0.162
	GG	302	390	1.00	–	–	1.00	–	–
rs17045754	CG	156	160	1.26	0.96–1.64	0.091	1.27	0.96–1.67	0.095
	CC	15	14	1.38	0.66–2.91	0.392	1.37	0.63–2.97	0.421
	TT	224	242	1.00	–	–	1.00	–	–
rs843720	GT	210	258	0.88	0.68–1.14	0.327	0.85	0.65–1.11	0.243
	GG	38	64	0.64	0.41–1.00	**0.048**	0.64	0.41–1.01	0.057
	TT	235	321	1.00	–	–	1.00	–	–
rs843645	GT	206	202	1.39	1.08–1.80	**0.011**	1.40	1.07–1.82	**0.014**
	GG	30	41	1.00	0.61–1.65	0.998	0.96	0.57–1.60	0.868
	CC	310	392	1.00	–	–	1.00	–	–
rs11125529	AC	149	159	1.19	0.91–1.55	0.216	1.20	0.91–1.58	0.204
	AA	13	13	1.27	0.58–2.77	0.557	1.16	0.52–2.60	0.718
	GG	297	377	1.00	–		1.00	–	
rs12615793	AG	160	173	1.17	0.90–1.53	0.233	1.18	0.90–1.55	0.243
	AA	15	14	1.36	0.65–2.86	0.418	1.21	0.56–2.60	0.630
	CC	126	178	1.00	–		1.00	–	
rs843711	TC	218	278	1.11	0.83–1.48	0.488	1.13	0.84–1.52	0.426
	TT	127	107	1.68	1.19–2.37	**0.003**	1.62	1.13–2.31	**0.008**
	CC	288	376	1.00	–		1.00	–	
rs11896604	GC	164	167	1.28	0.98–1.67	0.066	1.32	1.00–1.73	0.050
	GG	19	21	1.18	0.62–2.24	0.610	1.08	0.56–2.08	0.824
	CC	124	177	1.00	–		1.00	–	
rs843706	AC	219	277	1.13	0.84–1.51	0.414	1.14	0.84–1.53	0.404
	AA	128	108	1.69	1.20–2.39	**0.003**	1.62	1.13–2.31	**0.008**

*P*-values were calculated using the Wald test.

*P* < 0.05 indicates statistical significance.

The results of genetic model analyses (dominant, recessive, and additive) after adjustment for age and gender are presented in Table 4. We found that rs6713088 was associated with a 1.32-fold and 1.23-fold increased risk of liver cancer under the dominant model (OR = 1.32, 95% CI: 1.01–1.74, *P* = 0.043) and additive model (OR = 1.23, 95% CI: 1.02–1.48, *P* = 0.028), respectively. Additionally, rs843645 was associated with an increased risk of liver cancer under the dominant model (OR = 1.32, 95% CI: 1.02–1.70, *P* = 0.033). Rs843711 and rs843706 were associated with an increased risk of liver cancer under the recessive model (OR = 1.50, 95% CI: 1.11–2.03, *P* = 0.009; OR = 1.49, 95% CI: 1.10–2.02, *P* = 0.009, respectively) and additive model (OR = 1.26, 95% CI: 1.06–1.51, *P* = 0.010; OR = 1.26, 95% CI: 1.06–1.51, *P* = 0.010, respectively). In contrast, rs1682111 was associated with a decreased risk of liver cancer under both the dominant and additive model (OR = 0.68, 95% CI: 0.53–0.88, *P* = 0.003; OR = 0.75, 95% CI: 0.61–0.91, *P* = 0.004, respectively). Finally, rs843720 was associated with a decreased risk of liver cancer under the additive model (OR = 0.82, 95% CI: 0.68–1.00, *P* = 0.049).

**Table 4:** Genetic model analyses of the association between SNPs in *ACPY2* and the risk of liver cancer

SNP-ID	Dominant			Recessive			Additive		
	OR	95% CI	*P*	OR	95% CI	*P*	OR	95% CI	*P*
rs6713088	1.32	1.01–1.74	**0.043**	1.29	0.92–1.81	0.138	1.23	1.02–1.48	**0.028**
rs12621038	1.22	0.93–1.61	0.158	0.93	0.67–1.28	0.646	1.06	0.80–1.28	0.499
rs1682111	0.68	0.53–0.88	**0.003**	0.73	0.47–1.14	0.168	0.75	0.61–0.91	**0.004**
rs843752	1.21	0.94–1.56	0.135	1.03	0.65–1.65	0.886	1.13	0.93–1.38	0.218
rs10439478	1.23	0.94–1.61	0.130	1.17	0.84–1.64	0.351	1.15	0.96–1.38	0.125
rs17045754	1.28	0.97–1.67	0.077	1.27	0.59–2.74	0.536	1.24	0.97–1.57	0.080
rs843720	0.81	0.63–1.05	0.109	0.70	0.45–1.08	0.103	0.82	0.68–1.00	**0.049**
rs843645	1.32	1.02–1.70	**0.033**	0.83	0.50–1.37	0.471	1.16	0.94–1.42	0.158
rs11125529	1.20	0.91–1.57	0.197	1.10	0.49–2.45	0.818	1.16	0.91–1.48	0.226
rs12615793	1.18	0.90–1.54	0.224	1.15	0.53–2.45	0.728	1.15	0.91–1.46	0.238
rs843711	1.27	0.96–1.68	0.095	1.50	1.11–2.03	**0.009**	1.26	1.06–1.51	**0.010**
rs11896604	1.29	0.99–1.68	0.061	0.98	0.51–1.89	0.960	1.20	0.96–1.50	0.115
rs843706	1.28	0.96–1.69	0.090	1.49	1.10–2.02	**0.009**	1.26	1.06–1.51	**0.010**

*P*-values were calculated using the Wald test with adjustment for age and gender.

*P* < 0.05 indicates statistical significance.

**Figure 1:**
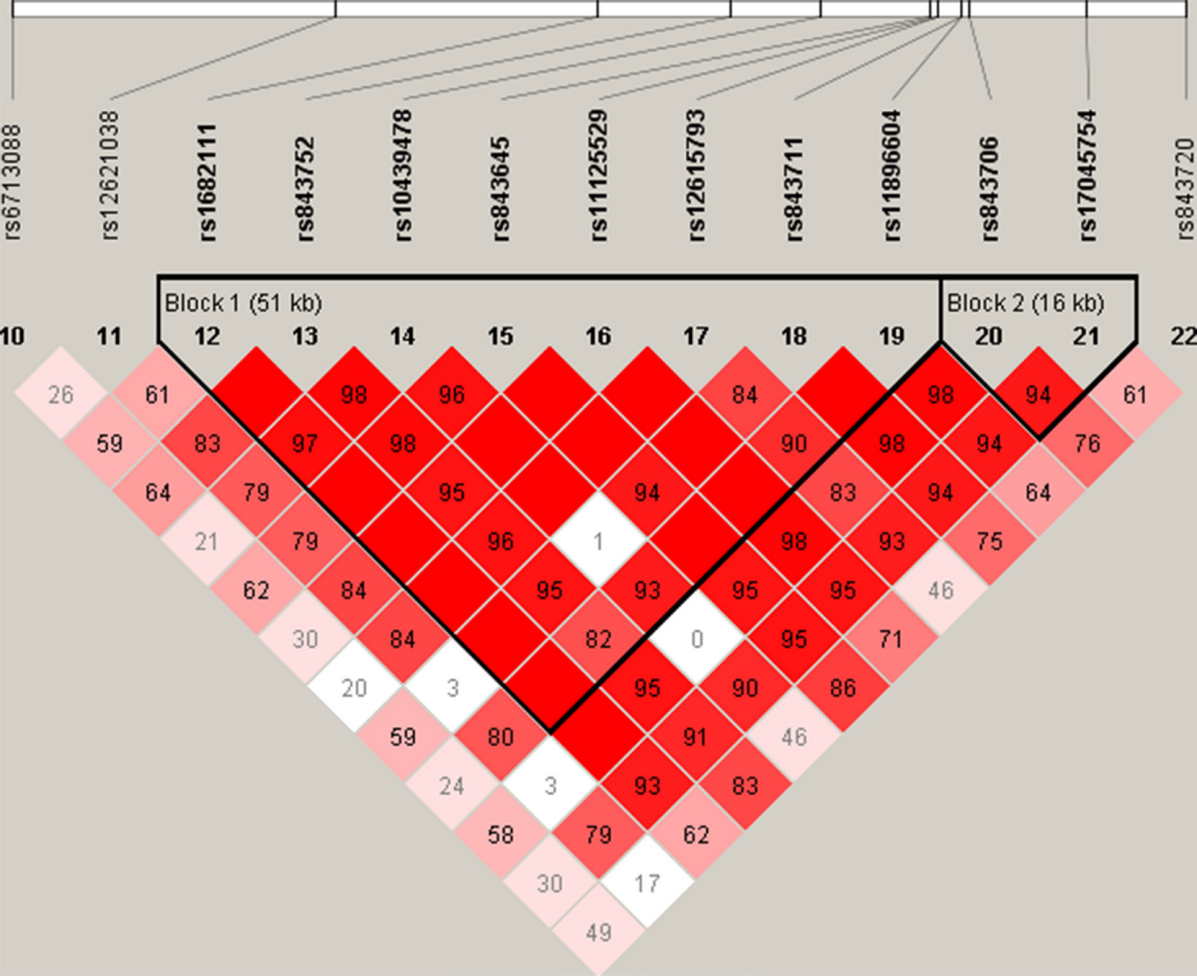
Linkage disequilibrium analysis of the 13 SNPs in *ACYP2* Block 1 includes rs1682111, rs843752, rs10439478, rs843645, rs11125529, rs12615793, rs843711, and rs11896604. Block 2 includes rs843706 and rs17045754.

We next analyzed the association between *ACYP2* haplotypes and the risk of liver cancer. We identified one linkage disequilibrium (LD) block consisting of eight SNPs (rs1682111, rs843752, rs10439478, rs843645, rs11125529, rs12615793, rs843711, and rs11896604). A second block consisting of two SNPs (rs843706 and rs17045754) also exhibited strong LD as shown in Figure 1. The ATATCGCC and CG haplotypes were associated with a decreased risk of liver cancer (OR = 0.76, 95% CI: 0.62–0.92, *P* = 0.006; OR = 0.78, 95% CI: 0.65–0.93, *P* = 0.006, respectively) (Table 5).

**Table 5:** Association between *ACYP2* haplotypes and the risk of liver cancer

SNPs	Haplotype	Without adjustment			With adjustment		
		OR	95% CI	*P*	OR	95% CI	*P*
rs1682111/ rs843752/ rs10439478/ rs843645/ rs11125529/ rs12615793/ rs843711/ rs11896604	ATATCGCC	0.78	0.64–0.94	**0.010**	0.76	0.62–0.92	**0.006**
	TTCTAATG	1.18	0.94–1.49	0.160	1.18	0.93–1.51	0.176
	TGAGCGTC	1.14	0.93–1.39	0.204	1.12	0.91–1.38	0.288
	TTCTCGCC	0.95	0.76–1.18	0.641	1.02	0.81–1.28	0.851
	TTCTCGTG	1.21	0.55–2.63	0.636	1.19	0.53–2.67	0.677
	TTCTCACC	0.9	0.43–1.88	0.789	0.81	0.38–1.72	0.587
rs843706/ rs17045754	AC	1.21	0.96–1.53	0.100	1.22	0.96–1.55	0.107
	AG	1.21	1.00–1.46	0.055	1.17	0.96–1.43	0.115
	CG	0.76	0.64–0.91	**0.002**	0.78	0.65–0.93	**0.006**

*P*-values were calculated using the Wald test.

*P* < 0.05 indicates statistical significance.

## DISCUSSION

We evaluated the relationships between 13 SNPs in *ACYP2* and the risk of liver cancer in a Han Chinese population. Our results indicated that rs6713088, rs843645, rs843711, and rs843706 were associated with an increased risk of liver cancer. In contrast, rs1682111, rs843720, the ATATCGCC haplotype (rs1682111, rs843752, rs10439478, rs843645, rs11125529, rs12615793, rs843711, and rs11896604), and the CG haplotype (rs843706 and rs17045754) were associated with a decreased risk of liver cancer.

We did not observe an association between rs11125529 and the risk of liver cancer. Previous studies have investigated the association between rs11125529 and coronary heart disease (CHD) [14], gastric cancer [15], high-altitude pulmonary edema (HAPE) [16], glioma [17], and head and neck squamous cell carcinoma [18]. However, no significant associations have been detected. Rs11125529 was associated with a decreased risk of breast cancer in a Han Chinese population based on a genotype model (AC vs. CC) [19], but it was associated with an increased risk of ischemic stroke [20]. A recent genome-wide meta-analysis found that rs11125529 in *ACYP2* was associated with telomere length [11]. Telomeres are critical for maintaining chromosomal stability and integrity [21–23]. They are approximately 10–15kb in human somatic cells and become progressively shorter by approximately 30–200 bp with each round of cell division as a result of incomplete DNA replication at the 3′ ends of chromosomes [24]. A reduction in telomere length to a critical threshold can lead to double-strand breaks, cell senescence, or apoptosis, which can accelerate human aging and death [21, 25]. Telomere length has been implicated in the development of multiple cancers, including hepatocellular carcinoma [12], lung [26], breast [27], bladder [28], and gastric [29], and so on.

We found that rs843711 and rs843706 were associated with an increased risk of liver cancer. rs843711 was reported previously associated with an increased risk of ischemic stroke [20], and similar results were obtained for rs843706, it was observed to be associated with an increased risk of breast cancer in a Han Chinese population [19]. In the study, no association was observed between the four SNPs (rs12621038, rs17045754, rs12615793, and rs11896604) and the risk of liver cancer. However, previous studies have demonstrated that rs12621038 and rs17045754 were associated with a decreased risk of breast cancer [30], but rs17045754 was found associated with an increased risk of ischemic stroke [20]. Rs12615793 and rs11896604 were discovered that them were associated with a decreased risk of HAPE [16], but the two SNPs were reported previously associated with an increased risk of ischemic stroke [20]. In addition, rs11896604 was reported previously associated with a decreased risk of breast cancer [19]. We found that rs6713088 was associated with an increased risk of liver cancer in the present study, and it was associated with an increased risk of ischemic stroke in a Han Chinese Population [20]. Simultaneously, we observed that rs1682111 was associated with a decreased risk of liver cancer in the study. However, it was reported previously associated with an increased risk of breast cancer [30] and lung cancer [31]. This phenomenon may be due to the impact of SNPs on different diseases is different. Interestingly, telomere length has been associated with liver cancer risk. Therefore, *ACYP2* polymorphisms may contribute to liver cancer through impacting telomere length.

Hepatocarcinogenesis is through that the genomic changes progressively alter the hepatocellular phenotype, thereby produce cellular intermediates, and ultimately develop into liver cancer [32]. The pathogenesis of liver cancer is considered a multistep and complex process. Previous study has shown that HBV and HCV infection often cause hepatitis, hepatic damage, and subsequent cirrhosis, eventually initiating liver carcinogenesis [33]. Inflammation is also closely associated with liver cancer initiation, progression, and metastasis. Inflammatory cells by releasing chemicals to induce peripheral cell mutation, and promoting the development of liver cancer [34]. Carcinogens (as pesticides) can promote spontaneous initiation, cytotoxicity with sustained cell proliferation, oxidative stress, formation of activated receptors and some others to increase the risk of liver cancer [35]. In addition, previous reports showed that multiple genetic and epigenetic changes are involved in the molecular pathogenesis of liver cancer, for example, somatic mutations in the p53 tumor suppressor gene (*TP53*) and the activation of the WNT signal transduction pathway [33, 36]. However, the pathogenesis of liver cancer has not been elucidated completely, and need to be further explored in the future.

Our study had several limitations. First, all study participants were of the Han Chinese population and our sample size was relatively small. Second, many other risk factors including smoking and alcohol consumption were not analyzed due to a lack of corresponding clinical data. Third, we did not perform any functional analyses. Finally, because our study is the first to report associations between *ACPY2* SNPs and liver cancer, larger and more comprehensive analyses of other patient populations must be performed to confirm our results.

In conclusion, our results demonstrate that rs6713088, rs843645, rs843711, and rs843706 are associated with an increased risk of liver cancer, and that rs1682111, rs843720, and the haplotypes (ATATCGCC and CG) are associated with a decreased risk of liver cancer in the Han Chinese. Our study provides theoretical basis for the prediction of liver cancer risk and the studies on the pathogenesis of liver cancer.

## MATERIALS AND METHODS

### Study participants

A total of 473 patients who were diagnosed with liver cancer and admitted to the Second People's Hospital of Hainan Province and the Agricultural Reclamation General Hospital of Hainan Province between May 2014 and June 2015 were enrolled in the study. The control group consisted of 564 randomly selected individuals from the health examination center of the Second People's Hospital of Hainan Province and the Agricultural Reclamation General Hospital of Hainan Province during the same period with no history of cancer or other diseases. All patients with the diagnosis of liver cancer should be confirmed by pathological examinations. All cases were verified, and the patients were recruited without any restrictions regarding age, sex, or disease stage. All subjects were unrelated Han Chinese whose ancestors had lived in the region for at least three generations. Patients with previous history of other cancers, cancer-related treatments, surgical contraindication, pregnancy, embryo-derived tumour, active liver disease, psychiatric history or poor compliance were excluded. The study protocol was approved by the Ethics Committee of the Second People's Hospital of Hainan Province and Northwest University, and was performed in accordance with the Declaration of Helsinki. Written informed consent was obtained from each patient prior to participation in the study.

### DNA extraction

We collected 5 mL of peripheral venous blood from each participant into vacutainer tubes containing ethylene diamine tetra-acetic acid. The blood samples were stored at −80°C until use. Genomic DNA was extracted from whole blood samples using the GoldMag-Mini Whole Blood Genomic DNA Purification Kit according to the manufacturer's protocol (GoldMag. Co. Ltd., Xi’an, China). DNA samples were stored at −4°C for future use. DNA concentration and purity were evaluated using a spectrophotometer (NanoDrop 2000; Thermo Fisher Scientific, Waltham, MA, USA).

### SNPs selection and genotyping

Thirteen SNPs (rs6713088, rs12621038, rs1682111, rs843752, rs10439478, rs17045754, rs843720, rs843645, rs11125529, rs12615793, rs843711, rs11896604, and rs843706) in *ACYP2* have been previously reported that are associated with several diseases and cancers risks, such as CHD [14], HAPE [16], ischemic stroke [20], breast [19, 30] and lung cancer [31], and the thirteen SNPs with a MAF > 5% in the HapMap of the Chinese Han Beijing (CHB) population were selected for genotyping. The PCR primers for each SNP were designed using the Sequenom MassARRAY Assay Design 3.0 Software (Sequenom, San Diego, CA, USA). Genotyping was performed using the Sequenom MassARRAY platform and the manufacturer's protocol. Data management and analysis were performed using the Sequenom Typer 4.0 software.

### Statistical analysis

All statistical analyses were performed using the Statistical Package for Social Sciences (SPSS, version 19.0) and Microsoft Excel. The genotype frequency distribution of the 13 SNPs in the controls was analyzed for deviations from HWE using Pearson's chi-square tests. Age, gender, allele, and genotype frequencies were compared between the cases and groups using chi-square tests/Fisher's exact tests. We analyzed the associations between SNPs in *ACPY2* and the risk of liver cancer under dominant, recessive, and additive genetic models using the PLINK software. We used the Haploview software package (version 4.2) platform for analyses of pairwise LD and haplotype structure. ORs and 95% CIs were calculated using unconditional logistic regression models and adjusted for age and gender. A *P*-value < 0.05 was considered statistically significant and all statistical tests were two-sided.

## SUPPLEMENTARY MATERIALS TABLE


