# Supplementary material for: ACYP2 polymorphisms are associated with the risk of liver cancer in a Han Chinese population
Source: Oncotarget. 2017 Jun 19;8(40):67723–31. doi: 10.18632/oncotarget.18574 (PMC5620206; doi:10.18632/oncotarget.18574)
Supplement: Supplementary file 1 [file oncotarget-08-67723-s001.pdf]

ACYP2 polymorphisms are associated with the risk of liver cancer in a Han Chinese population

Supplementary Materials

Supplementary Table 1: The PCR and UE primers of loci used in this study

SNP-ID	2nd-PCRP	1st-PCRP	UEP-SEQ
rs6713088	ACGTTGGATGGTCACCAAAACACGTAATG	ACGTTGGATGACACACACAGACTCCTTCAC	gaggcCAGAATGGTCCACTAGAGA
rs12621038	ACGTTGGATGGGCATAAGTTTTATTGCCTC	ACGTTGGATGATTGTGCTAGGCACTTTAGG	ccATTGCCTCAGCTAGACT
rs1682111	ACGTTGGATGGCCAGTGGGAATGCAAAATG	ACGTTGGATGGAATTGCTGGGTTATTTGGC	tgtcATGCAAAATGAAACAGACACTT
rs843752	ACGTTGGATGGAGACAACATAATGGAGGTC	ACGTTGGATGTCCTCTTTTCAGAAACCTGC	cGAGTTTGGGTTTGAGGT
rs10439478	ACGTTGGATGCTACACTCTCCAGAGGAATG	ACGTTGGATGTAGCACAAGACCTACACTGG	TTGCTGTTTTCCCAGAA
rs17045754	ACGTTGGATGGAAATCAGGGATATTAGTGC	ACGTTGGATGCTGTAAAAGTTCTGGCATGG	caggTATTCAGCTTCCTAGAGTTA
rs843720	ACGTTGGATGAGTCAGAGCTAGACCTCTGG	ACGTTGGATGCTTCACAACACTCCTGTAAG	ccccAATCTGTCTCAGGGTCTT
rs843645	ACGTTGGATGACAGTGCCTTTAGCAAGGTG	ACGTTGGATGGAAATCTGAATACCACCTAC	TCATAGGCACTACTGTATC
rs11125529	ACGTTGGATGCCGAAGAAAAGAAGATGAC	ACGTTGGATGGAGCTTAGTTGTTTACAGATG	AGAAAAGAAGATGACTAAAACAT
rs12615793	ACGTTGGATGATCTTGGCCCTTGAAGAA	ACGTTGGATGTTTGAGCTTAGTTGTTTAC	AAATTGAGTGACAAATATAAACTAC
rs843711	ACGTTGGATGTGCCTTGTGGGAATTAGAGC	ACGTTGGATGGACAAAGGACCTTACAACTC	gggaTCAGGGAACCAGTGCAAA
rs11896604	ACGTTGGATGTGTCTCTGACCTAGCATGTA	ACGTTGGATGAAGTCAGAATAGTGCTTAC	GTTAAGCTTGCAAGGAG
rs843706	ACGTTGGATGTGAATAACTTGGTCTTATC	ACGTTGGATGTGAAAGCCATAAATATTTTG	cACTTGGTCTTATCTGATGC

SNP: Single nucleotide polymorphism; PCRP: Polymerase chain reaction primer; UEP: unique base extension primer.
Sequences are written in the 5′→3′ (left to right) orientation.
